# Health-economic benefits of anti-CD20 treatments in relapsing multiple sclerosis estimated using a treatment-sequence model

**DOI:** 10.1177/20552173231189398

**Published:** 2023-07-24

**Authors:** Ide Smets, Matthijs Versteegh, Simone Huygens, Cato Corsten, Beatrijs Wokke, Joost Smolders

**Affiliations:** Department of Neurology, 6993 Erasmus Medical Center, Rotterdam, The Netherlands; Huygens & Versteegh, Zwijndrecht, The Netherlands; Department of Neurology, 6993 Erasmus Medical Center, Rotterdam, The Netherlands

**Keywords:** Multiple sclerosis, disease-modifying treatment, anti-CD20 monoclonal antibodies, cost-effectiveness research, net health benefit, quality-adjusted life years

## Abstract

**Background:**

In high-income countries, four anti-CD20 monoclonal antibodies (mAbs) are used or in the pipeline for relapsing MS: ocrelizumab, ofatumumab (both registered), ublituximab (awaiting registration) and rituximab (off-label). List prices differ significantly between registered and off-label drugs.

**Objective:**

Comparing differences in benefits between anti-CD20 mAbs from a health-economic and societal perspective.

**Methods:**

To reflect lifetime use of DMTs, we used a treatment-sequence model to compare ocrelizumab/ofatumumab and eight other drug classes in terms of health (lifetime relapses, time to Expanded Disability Status Scale [EDSS] 6, lifetime quality-adjusted life years) and cost-effectiveness (net health benefit). To become cost-effective compared to ocrelizumab, we modelled the list price of ublituximab and desired effect on EDSS progression of rituximab.

**Results:**

Although drug sequences with ocrelizumab in first- and second-line were more cost-effective than ofatumumab, our probabilistic analysis suggests this outcome was very uncertain. To be more cost-effective than ocrelizumab, ublituximab needs to be about 25% cheaper whilst rituximab needs to equal the effect on disability progression seen with first-line treatments.

**Conclusions:**

Our model showed no clear difference in cost-effectiveness between ocrelizumab and ofatumumab. Hence, prescribing the least costly anti-CD20 mAb can democratise MS care without a loss in health benefits.

## Introduction

Over the last years, the MS field has become accustomed to the sequential introduction of new agents from the same drug class, such as interferons, fumarates, sphingosine-1-phosphate receptor (S1PR) modulators, cladribine, and anti-CD20 monoclonal antibodies (mAbs).^1^ The expanding possibilities for treatment of people with MS (pwMS) and increase in the use of high-efficacy therapies earlier in the disease course result in a continuous increase of the costs of treatment.^2,3^ However, health economic considerations are not necessarily part of standard MS care. Four different anti-CD20 mAbs (ocrelizumab, ofatumumab, ublituximab, rituximab) have been shown to be effective in treating pwMS. However, to what degree biological differences translate into clinical ones remains incompletely understood. All anti-CD20 mAbs deplete peripheral B cells to undetectable levels.^4^ Initially, they have been positioned as a second-line therapy. However, evolving insights regarding the importance of early high-efficacy treatment as well as their favourable safety profile, have shifted their use in pwMS towards the first line.^5,6^ Based on the experience in rheumatic diseases, rituximab was the first anti-CD20 mAb explored for use in MS.^7^ Although the primary outcomes were met in its phase 2 trial,^7^ commercial development was abandoned. Less immunogenic anti-CD20 mAbs were subsequently developed as MS treatments.^8,9^ Nonetheless, many countries use rituximab off-label and experiences are on the whole positive.^6,10–12^ Ocrelizumab is the only anti-CD20 mAb with approval for use in primary progressive disease,^13^ and ofatumumab has a unique mode of administration as it can be self-injected subcutaneously. The phase 3 trial of ublituximab was recently published, and the drug is awaiting registration.^14^ Ublituximab is another potent B cell-depleting agent reducing infusion time. The lack of head-to-head studies between anti-CD20 mAbs and the use of different active comparators preclude direct comparison of efficacy and safety in an already potent therapeutic class.^4,15^ Biologically, differences in B cell repopulation kinetics are seen but as yet of unknown clinical relevance.^4^ Therefore, differences in the clinical use of these drugs are based on practical considerations, such as side effects, considerations around pregnancy, and route of administration, among others.^4,15^ Although a shift towards higher priced disease-modifying therapies (DMTs) substantially increases the economic burden of MS, the wider health-economic implications are only sparsely appreciated in the DMT selection process.^2,3,16^ Hence, there is a societal need to harmonise pricing of drugs with similar health benefit profiles allowing them to become an important criterion for treatment selection. Using Dutch DMT list prices, we used a health-economic approach to differentiate anti-CD20 mAbs in terms of health benefits (relapse rate reduction, disability prevention), direct DMT costs, alongside indirect and societal costs for the care of people with relapsing MS. To be cost-effective compared to sequences with ocrelizumab, we subsequently estimated the optimal list price of ublituximab and minimally desired effect of rituximab on disability progression.

## Methods

### Erasmus MC/iMTA ms model

We used a previously published treatment sequence model, the ErasmusMC/iMTA MS, model to compare DMT sequences.^17,18^ The model estimates health outcomes (i.e., lifetime relapses, time to Expanded Disability Status Scale [EDSS] 6), lifetime quality-adjusted life years [QALYs]) and cost-effectiveness (i.e., net health benefit [NHB]). Cost accrual over a patient's lifetime included DMT costs, DMT monitoring costs, administration costs, in-patient stay, costs related to relapses as well as societal costs such as loss of productivity and caregiver time. The key features of the model are treatment switches based on clinical decision rules and DMT sequences of up to five treatments. In an escalation setting, first-line treatments are divided into line 1a and line 1b. Line 1b is for those who experience adverse events on line 1a. If high-efficacy treatments are used as first line, line 1b is less relevant and was removed. We refer to the subsequent treatment lines as lines 2, 3 and 4. PwMS transition through these lines if they experience an event, that is, a relapse, disease progression based on EDSS steps or adverse events. The likelihood of these events occurring is estimated using a separate network meta-analyses (NMA) as described elsewhere.^19^ PwMS aged 50 years or older with at least 5 years without relapse or EDSS progression, who were on their first- or second-line treatment, and pwMS on third-line treatment at the age of 70 years or older with at least 10 years without relapse or EDSS progression discontinued DMTs.

### DMT sequences

In the European Union, typically 12 DMTs are used for the treatment of relapsing MS, 13 when including the off-label use of rituximab, and 14 when anticipating the potential European Medicine Agency approval of ublituximab. These DMTs include interferon beta (INFB), dimethyl fumarate 240 mg (DMF), teriflunomide 14 mg (TER), glatiramer acetate 20 mg (GLA), ponesimod 20 mg (PON), ozanimod 1 mg (OZA), fingolimod 0.5 mg (FIN), natalizumab 300 mg (NAT), cladribine 3.5 mg/kg (CLA), ocrelizumab 600 mg (OCR), ofatumumab 20 mg (OFA), rituximab 500/1000 mg (RIT), ublituximab 450 mg (UBL) and alemtuzumab 12 mg (ALE). In an escalation setting, first-line treatments consist of INFB, DMF, TER, GLA, FIN, PON and OZA. As there is a trend towards starting high-efficacy treatments earlier in the disease course, we also modelled anti-CD20 mAbs as first-line treatments. Alemtuzumab was always restricted to the last line. We only included clinically plausible DMT switches (i.e., excluding switches with the same mode of action such as prescribing one anti-CD20 mAb after another) based on clinical practice in The Netherlands. The model assumes that each sequence has an equal likelihood of being used in clinical practice.

### Efficacy and costs of anti-CD20 mAbs

Table 1 describes the estimates of the NMA for relapses and 6-month confirmed disability progression (CDP) for four anti-CD20 mAbs. Ublituximab efficacy is taken from the NMA but its list price is still unknown. Rituximab was included using relapse rate data from the earlier phase 2 trial (RIT 1000 mg)^7^ and the recent RIFUND-MS trial (RIT 500 mg).^20^ We ran the analyses using the cost for both 500 and 1000 mg. Only the RIFUND-MS trial reported on rituximab's effect on EDSS progression, but this trial was small (n = 195) implying large confidence intervals making them less suitable for this analysis. The drug acquisition costs of DMTs were based on 2022 list prices in The Netherlands,^21^ and do not take into account discounts provided by pharmaceutical companies to hospitals.

**Table 1.** Efficacy as estimated in the network meta-analysis and Dutch costs of anti-CD20 mAbs.

DMT	Incidence rate ratio of ARR vs. placebo [95% CI]	Relative risk of 24-week CDP vs. placebo [95% CI]	Annual Dutch drug acquisition costs (Euros)	
			First year	Subsequent years
OCR	0.40 [0.30–0.52]	0.45 [0.31–0.61]	22,437	22,437
OFA	0.30 [0.22–0.42]	0.55 [0.34–0.88]	23,434	20,086
UBL	0.31 [0.21–0.45]	0.54 [0.27–1.08]	NA	NA
RIT	0.36 [0.20–0.64]	NA	3,802	2,535

Details on the network meta-analysis can be found are described elsewhere.^19^ As the trial of rituximab was not powered for 6-month CDP, we consider the ability of rituximab to reduce disease progression to be unknown in our analyses. For amounts in Euro integers were used. ARR: annualized relapse rate; CDP: confirmed disability progression; CI: confidence interval; DMT: disease-modifying treatment; NA: not applicable; OCR: ocrelizumab; OFA: ofatumumab; RIT: rituximab; UBL: ublituximab.

### Health economic analyses

We ran the microsimulation model with 10,000 treatment-naïve relapsing pwMS per DMT sequence, with an assumed starting age of 29 and evenly distributed at diagnosis over EDSS class 0 to 3. All costs and effects were discounted with 4% and 1.5%, respectively, and a Dutch societal perspective was adopted.^22^ Using these model settings, we conducted three analyses.

In the main analyses, we compared the cost-effectiveness of ocrelizumab and ofatumumab as first- and second-line treatments. Assuming that after anti-CD20 mAbs as first-line only other high-efficacy treatments would be provided (CLA, NAT, ALE) and that alemtuzumab was restricted to being a last-resort DMT, there are four possible sequences in four treatment lines (OCR/OFA-CLA-NAT-ALE or OCR/OFA-NAT-CLA-ALE). If anti-CD20 mAbs are used as second-line, there are 144 possible sequences for ocrelizumab and ofatumumab taking into account all potential preceding and following treatment lines, including those in line 1b. We conducted probabilistic sensitivity analyses (PSA) to explore uncertainty, simultaneously drawing from the confidence intervals of the model’s input parameters. As this analysis is more computationally intensive, we conducted 500 model iterations for a cohort of 1000 pwMS. For the PSA, we compared ocrelizumab and ofatumumab in sequences that ranked highest in terms of NHB. In a second analysis, we estimated at which annual drug cost ublituximab would have a similar NHB as the most cost-effective sequence in the first- and second-line. In a third analysis, we estimated which relative risk reduction versus placebo would be required for rituximab 500 mg in future trials powered for 6-month CDP to have a similar NHB as the most cost-effective sequence in the first- and second-line.

### Definitions

*Utility* represents how bad a certain state of health feels for the general public based on questionnaires. Utility values (and thus QALYs) decrease when pwMS progress through EDSS scores, experience relapses or adverse events.^23^

*QALY* is a combined metric of length and quality of life. Life years are estimated with survival analysis and quality of life by asking patients to fill out preference-based questionnaires (utility). Living for 10 years in EDSS 2 without relapses yields 0.782*10 = 7.82 QALYs.

*NHB* expresses the cost-effectiveness of treatment sequences. It uses the monetary value of a QALY (€50,000 for MS in The Netherlands) to express the costs in terms of QALYs.^18^ NHB is the total QALYs gained by a treatment sequence minus the value in terms of lost QALYs due to the costs required to generate those QALYs. In a formula: QALYs – (costs/value of a QALY).

## Results

### Cost-effectiveness of ocrelizumab versus ofatumumab as first- and second-line treatments

**Figure 1.**
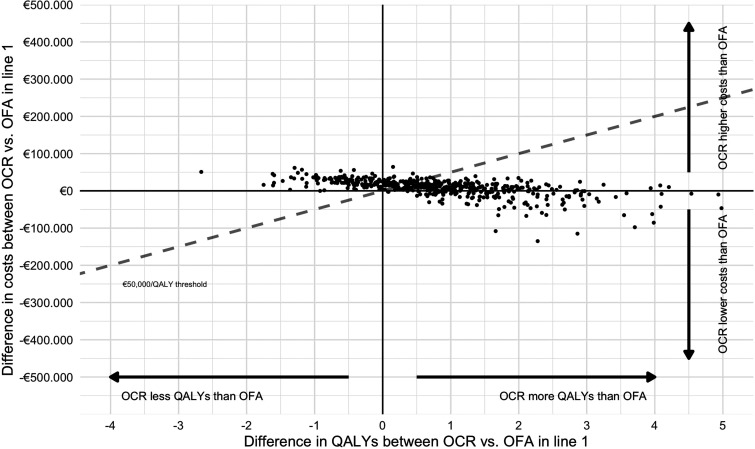
Probabilistic sensitivity analysis of ocrelizumab versus ofatumumab as a first-line treatment. Results were modelled for ocrelizumab/ofatumumab followed by cladribine-natalizumab-alemtuzumab. The dashed line represents the willingness-to-pay threshold of €50,000/QALY. OCR: ocrelizumab; OFA: ofatumuab; QALY: quality-adjusted life year.

**Figure 2.**
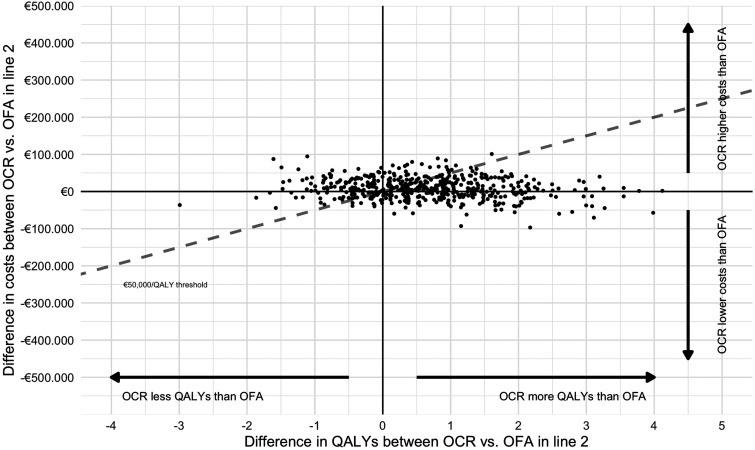
Probabilistic sensitivity analysis of ocrelizumab vs. ofatumumab as a second-line treatment. Results were modelled for interferon-beta-glatiramer acetate-ocrelizumab/ofatumumab-cladribine-alemtuzumab. The dashed line represents the willingness-to-pay threshold of €50,000/QALY. OCR: ocrelizumab; OFA: ofatumuab; QALY: quality-adjusted life year.

Table 2 shows the results of the deterministic analyses of sequences with currently registered anti-CD20 mAbs ocrelizumab or ofatumumab as first- or second-line treatment. In both modelled treatment lines, ocrelizumab yields more QALYs than ofatumumab. This is driven by its larger effect on 6-month CDP, even though ofatumumab is better in prevention of relapses. Treatment with ocrelizumab does come at a higher cost than treatment with ofatumumab. The level of uncertainty regarding these findings is illustrated through the PSA (Table 3, Figures 1 and 2). These figures demonstrate that while our deterministic analysis showed larger QALY gains for ocrelizumab versus ofatumumab, there is considerable uncertainty. When varying input parameters, 24.2% and 27.0% of the iterations of the PSA resulted in ocrelizumab yielding less QALYs than ofatumumab in line 1 and 2, respectively. This suggests that the deterministic estimate which favoured ocrelizumab over ofatumumab in terms of QALY gains is reversed in about a quarter of the iterations. At a ‘willingness-to-pay’ of €50,000 per QALY, this translates into a relatively low probability of ocrelizumab being cost-effective compared to ofatumumab of 65% and 64% for lines 1 and 2, respectively.

**Table 2.** Mean cost-effectiveness outcomes of registered anti-CD20 mAbs.

Class	First line				Second line	
Combined by DMT	OCR-CLA-NAT-ALE	OCR-NAT-CLA-ALE	OFA-CLA-NAT-ALE	OFA-NAT-CLA-ALE	OCR	OFA
Total costs (Euros)	494,703	531,115	482,249	522,140	521,797	511,369
Drug costs (Euros)	290,094	329,325	269,390	311,977	297,984	281,995
Other healthcare costs (Euros)	127,820	126,629	130,580	129,484	135,678	137,480
Societal costs (Euros)	76,789	75,190	82,279	80,659	88,136	91,894
Total QALYs	20.4	20.6	19.7	19.9	19.2	18.5
NHB	10.55	10.0	10.0	9.4	8.7	8.3
Lifetime relapses	3.9	3.8	3.5	3.4	4.3	4.0
Time to EDSS 6 (years)	26.6	27.0	24.7	25.3	23.7	22.3
Time in line 1 (years)	9.9	10	11.1	11.2	NA	NA

The cost-effectiveness outcomes in second-line result from combining all sequences by the anti-CD20 mAb in second line divided by the total number of sequences. For amounts in Euro integers were used. DMT: disease-modifying treatment; OCR: ocrelizumab; OFA: ofatumumab; CLA: cladribine; NAT: natalizumab; ALE: alemtuzumab; QALY: quality-adjusted life year; NHB net health benefit; EDSS: Expanded Disability Status Scale; NA: not applicable.

**Table 3.** Mean cost-effectiveness outcomes of probabilistic sensitivity analysis.

	Line 1		Line 2	
DMT sequence	OCR-CLA-NAT-ALE	OFA-CLA-NAT-ALE	INFB-GLA-OCR-CLA-ALE	INFB-GLA-OFA-CLA-ALE
Total costs in Euros ± S.E. [CI]	487,715 ± 91,585 [138, 293–668, 307]	479,019 ± 97,562 [239, 287, 798–670]	449.723 ± 102,988 [247, 587, 867–651]	441,362 ± 106,262 [089–649, 233, 634]
Total QALYs ± S.E. [CI]	20.2 ± 1.2 [17.9–22.5]	19.4 ± 1.4 [16.7–22.1]	19.1 ± 1.2 [16.9–21.4]	18.5 ± 1.3 [16.0–21.0]

DMT: disease-modifying treatment; OCR: ocrelizumab; OFA: ofatumumab; CLA: cladribine; NAT: Natalizumab; ALE: alemtuzumab; IFNB: interferon-beta; GLA: glatiramer acetate; QALY: quality-adjusted life year; NHB: net health benefit; EDSS: Expanded Disability Status Scale; S.E.: standard error; CI: confidence interval.

### Cost-effectiveness analysis of ublituximab with estimated list price

As ublituximab is awaiting EMA registration, its list price is unknown and the NHB cannot be calculated. If sequences with ublituximab would have a similar NHB as those with ocrelizumab in the first-line, its annual drug costs should be at least 21.6% lower than ocrelizumab. In the Netherlands, this would result in an annual cost of €17,600 for ublituximab to have the same probability as ocrelizumab to be cost-effective. The price reduction is needed to offset the lower mean efficacy of ublituximab in preventing disability progression (Table 1). At that price, using ublituximab would result in €245,984 lifetime drug costs, €458,079 total costs and 19.7 QALY's. In second line, ublituximab's annual drug costs should be at least 22.4% lower than those of ocrelizumab, thus not exceeding €17,400. At that price, using ublituximab in the second line would result in €198,643 lifetime drug costs, €425,222 total costs and 18.7 QALY's.

### Estimated relative risk of disability progression for rituximab to be cost-effective

The effect of rituximab on disability progression in MS is poorly identified making it impossible to calculate the NHB. To equal the NHB of sequences with ocrelizumab as a first-line (i.e., OCR-CLA-NAT-ALE), the relative risk of rituximab for 6-month CDP versus placebo would have to be at least 0.80 (500 mg) and 0.75 (1000 mg). This means rituximab can be less performant in terms of disability reduction than ocrelizumab, as this would be offset by the lower list price of rituximab. At that relative risk, using rituximab would cost 24.6% less than ocrelizumab (in total €373,106 of which €139,270 are drug costs) to gain 18.1 QALYs (compared to 20.4 QALYs with ocrelizumab). If ocrelizumab is used as a second-line (i.e., INFB-GLA-OCR-CLA-ALE), rituximab's relative risk versus placebo for 6-month CDP would have to be 0.83 (500 mg) and 0.78 (1000 mg). At that relative risk, using rituximab would cost 26.6% less than ocrelizumab (in total €333,479 of which €89,866 are drug costs) to gain 17.0 QALYs (compared to 19.3 QALYs with ocrelizumab). Although less QALYs are gained, the lower costs of DMT sequences including rituximab results in an equal NHB at these relative risks for 6-month CDP as the same sequences with ocrelizumab in first- or second-line.

## Discussion

In this health economic modelling study, we showed that there is no clear difference in the cost-effectiveness of sequences with ocrelizumab and ofatumumab in either first- or second-line in relapsing MS. The probability of ocrelizumab being cost-effective versus ofatumumab in first- and second-line based on our model is about 65% and, thus, only slightly better than what would be expected from chance. Moreover, we estimated that ublituximab should not be priced at more than 75% of ocrelizumab's list price to have an equal probability at cost-effectiveness. This contrasts with our cost-effectiveness analysis on S1PR modulators in which differences in health outcomes could not be offset by lower drug costs.^19^ Of note, ublituximab's higher efficacy to prevent relapses was not enough to compensate for the lower ability in reducing disease progression. This is caused by the larger impact of disease progression on both health care use (i.e., costs) and quality of life than relapses, which only have a temporary effect on both outcomes. From a health economic perspective, lower costs of treatment can compensate reduced efficacy whereas in the clinical community, this principle is controversial. The more costly treatment may thus not yield sufficiently more health to be worth the opportunity costs of spending this money elsewhere generating even more health. We acknowledge that cost-effectiveness studies can have an upward effect on pricing, by pricing towards the cost-effectiveness threshold. However, we also expect that market entry of new anti-CD20 mAbs would result in price competition and therefore report on ublituximab's cost-effective price proportional to ocrelizumab.

Our model showed that rituximab would already be the most cost-effective anti-CD20 mAb if its efficacy on 6-month CDP is comparable to traditional first-line therapies such as interferon-beta. Unfortunately, precise estimates of rituximab's effect on disability progression are lacking. There is also little prospect on collecting this data as this is entirely dependent on the commitment of a commercial sponsor. The fact that the RIFUND-MS trial only included 198 pwMS whereas industry-sponsored phase 3 trials with different anti-CD20 mAbs included between 1094 and 1882 individuals underpins this argument.^8,9,14,20^ In terms of preventing focal inflammation, our NMA estimates rituximab to be right in the middle between ublituximab and ocrelizumab. This confirms earlier data from its phase 2 trial and evidence from real-world comparative effectiveness studies that included pwMS with high risk of disease breakthrough.^6,7,11^ Along these lines, a large meta-analysis including 13,500 pwMS from different phase 3 trials showed that 61% of the effect on sustained disability could be explained by the effect on the accumulation of MRI lesions.^24^ Furthermore, rituximab has an established safety record and existing evidence does so far not support a clinically relevant role of anti-drug-antibodies.^12,25^ Although regulatory bodies should maintain high standards, the estimates on rituximab's cost-effectiveness are thought provoking and prompt the MS field and regulators to reconsider its use as a first-line treatment. Similar logic applies to subcutaneous cladribine which is a biosimilar of oral cladribine and comes at a significantly lower cost.^26^ Also, it would be worth considering to invest public funds in comparative research in which 6-month CDP is the primary endpoint.

It is important to acknowledge that the cross-trial comparisons which underpin our model are difficult to interpret because of the variability within trial populations. For example, ofatumumab (ASCLEPIOS) and ocrelizumab (OPERA) had different active comparators in their phase 3 trials.^8,9^ People from white Eastern European ancestry are overrepresented in more recent phase 3 trials.^8,9,14,27^ As MS trials are powered on annualised relapse rates but not disability endpoints, more variation on the latter is expected. This yields unexpected results such as ublituximab (ULTIMATE) not reaching its 6-month disability endpoint which lends weight to earlier observations of the rather modest effect of other anti-CD20 mAbs on slowing brain volume loss compared to active, but less potent, reference DMTs.^8,9,14^ Correspondingly, our PSA analysis showed a large overlap in confidence intervals surrounding the point estimates of relapse rate reduction and disability accrual not showing meaningfully different probabilities of cost-effectiveness between DMT sequences with ocrelizumab or ofatumumab. Illustratively, the event rates in the original phase 3 trials for 6-month disability progression were nearly identical in ULTIMATE (hazard ratio 0.66, p > 0.05) and ASCLEPIOS (hazard ratio 0.61, p = 0.01) against the same active comparator. Both trials were slightly outperformed by OPERA with a hazard ratio of 0.60 against interferon-beta.^8^ To what extent these differences in phase 3 trials impact on real-world outcomes, thereby justifying differences in list price, remains elusive. The lack of appropriate dose-finding studies across anti-CD20 mAbs feeds this uncertainty. Different B cell repopulation kinetics after administration suggest different depths of tissue depletion which might have ramifications for preventing disability progression.^4,15^ Dose-response relationships beyond no evidence of inflammatory disease targets could help to prioritise in the future.^28–30^ Cost-effectiveness of anti-CD20 mAbs may alter due to extended-dosing schedules based on B cell counts and risk mitigation strategies for chronic B cell depletion.^31–33^

In this study, we only compared different anti-CD20 mAbs while the cost-effectiveness of a first-line treatment also competes with other, cheaper DMTs. Moreover, we did not consider patient and prescriber's preferences regarding mode of administration (i.e., subcutaneous vs. intravenous) nor MRI data due to heterogeneity across radiological outcomes. Furthermore, price reductions following negotiations between pharmacies, hospitals and insurance companies are typically undisclosed but change national versus local cost-effectiveness. Nonetheless, societal expenses will remain the same as these discounts represent a transfer of profit from a pharmaceutical company to a hospital. Our NMA did not correct for the somewhat different criteria for measuring 6-month CDP in the OPERA and ASCLEPIOS trials.^8,9^ Another study addressed this issue by using ‘OPERA-aligned criteria’ finding small differences in favour of ofatumumab albeit with large overlapping confidence intervals.^34^ Alternatively, we underpinned our main conclusions by a PSA, which takes these overlapping intervals into account. The absence of a universally accepted PSA cut-off allows for other researchers to draw different conclusions on similar results.^35^ Finally, this study's results for the Dutch setting may not be transferable to other countries due to country-specific DMT list prices and switching practices. Nevertheless, the principles of weighing benefits of treatments against their costs are not and are of increasing relevance with the continuously rising costs of MS treatment.

In summary, cost-effectiveness is valuable as a third pillar in DMT decisions next to efficacy and safety when different compounds of the same therapeutic class are available. As there is extensive overlap in terms of health outcomes across registered, soon-to-be registered and off-label anti-CD20 mAbs, it would be justified to prescribe the least costly anti-CD20 mAb arguing for strong procurement practices in the interest of equity in MS care.

## References

[1] Cross A Riley C . Treatment of multiple sclerosis. Continuum (Minneap Minn) 2022; 28: 1025–1051. 35938656 10.1212/CON.0000000000001170

[2] Callaghan BC Reynolds E Banerjee M , et al. Out-of-pocket costs are on the rise for commonly prescribed neurologic medications. Neurology 2019; 92: E2604–E2613. 31043472 10.1212/WNL.0000000000007564 PMC6556089

[3] Khakban A Llorian ER Michaux KD , et al. Direct health care costs associated with multiple sclerosis: a population-based cohort study from 2001 to 2020 in British Columbia, Canada. Neurology 2023; 100: e899–e910. DOI:10.1212/WNL.0000000000201645 PMC9990437 36450607

[4] Bar-Or A O’Brien SM Sweeney ML , et al. Clinical perspectives on the molecular and pharmacological attributes of anti-CD20 therapies for multiple sclerosis. CNS Drugs 2021; 35: 985–997. 34370283 10.1007/s40263-021-00843-8 PMC8351586

[5] He A Merkel B Brown JWL , et al. Timing of high-efficacy therapy for multiple sclerosis: a retrospective observational cohort study. Lancet Neurol 2020; 19: 307–316. 32199096 10.1016/S1474-4422(20)30067-3

[6] Spelman T Magyari M Piehl F , et al. Treatment escalation vs immediate initiation of highly effective treatment for patients with relapsing-remitting multiple sclerosis: data from 2 different national strategies. JAMA Neurol 2021; 78: 1197–1204. 34398221 10.1001/jamaneurol.2021.2738 PMC8369379

[7] Hauser SL Waubant E Arnold DL , et al. B-cell depletion with rituximab in relapsing-remitting multiple sclerosis. N Engl J Med 2008; 358: 676–688. 18272891 10.1056/NEJMoa0706383

[8] Hauser SL Bar-Or A Comi G , et al. Ocrelizumab versus interferon Beta-1a in relapsing multiple sclerosis. N Engl J Med 2017; 376: 221–234. 28002679 10.1056/NEJMoa1601277

[9] Hauser SL Bar-Or A Cohen JA , et al. Ofatumumab versus teriflunomide in multiple sclerosis. N Engl J Med 2020; 383: 546–557. 32757523 10.1056/NEJMoa1917246

[10] Ineichen BV Moridi T Granberg T , et al. Rituximab treatment for multiple sclerosis. Mult Scler J 2020; 26: 137–152. 10.1177/1352458519858604 31237800

[11] Alping P Frisell T Novakova L , et al. Rituximab versus fingolimod after natalizumab in multiple sclerosis patients. Ann Neurol 2016; 79: 950–958. 27038238 10.1002/ana.24651

[12] Salzer J Svenningsson R Alping P , et al. Rituximab in multiple sclerosis: a retrospective observational study on safety and efficacy. Neurology 2016; 87: 2074–2081. 27760868 10.1212/WNL.0000000000003331 PMC5109942

[13] Montalban X Hauser SL Kappos L , et al. Ocrelizumab versus placebo in primary progressive multiple sclerosis. N Engl J Med 2017; 376: 209–220. 28002688 10.1056/NEJMoa1606468

[14] Steinman L Fox E Hartung H-P , et al. Ublituximab versus teriflunomide in relapsing multiple sclerosis. N Engl J Med 2022; 387: 704–714. 36001711 10.1056/NEJMoa2201904

[15] Cotchett KR Dittel BN Obeidat AZ . Comparison of the efficacy and safety of anti-CD20 B cells depleting drugs in multiple sclerosis. Mult Scler Relat Disord 2021; 49: 102787. 33516134 10.1016/j.msard.2021.102787 PMC9246073

[16] Bebo B Cintina I Larocca N , et al. The economic burden of multiple sclerosis in the United States: estimate of direct and indirect costs. Neurology 2022; 98: E1810–E1817. 35418457 10.1212/WNL.0000000000200150 PMC9109149

[17] Versteegh MM Huygens SA Wokke BWH , et al. Effectiveness and cost-effectiveness of 360 disease-modifying treatment escalation sequences in multiple sclerosis. Value Health 2022; 25: 984–991. 35667786 10.1016/j.jval.2021.11.1363

[18] Huygens S Versteegh M . Modeling the cost-utility of treatment sequences for multiple sclerosis. Value Health 2021; 24: 1612–1619. 34711361 10.1016/j.jval.2021.05.020

[19] Corsten C Huygens S Versteegh M , et al. Benefits of sphingosine-1-phosphate receptor modulators in relapsing MS estimated with a treatment sequence model. medRxiv 2022: 2022.12.23.22283885. 10.1016/j.msard.2023.105100 37944195

[20] Svenningsson A Frisell T Burman J , et al. Safety and efficacy of rituximab versus dimethyl fumarate in patients with relapsing-remitting multiple sclerosis or clinically isolated syndrome in Sweden: a rater-blinded, phase 3, randomised controlled trial. Lancet Neurol 2022; 21: 693–703. 35841908 10.1016/S1474-4422(22)00209-5

[21] Zorginstituut Nederland. Farmacotherapeutisch Kompas, https://www.farmacotherapeutischkompas.nl (2022, accessed 7 July 2022).

[22] Zorginstituut Nederland. Guideline for conducting economic evaluations in healthcare [in Dutch: Richtlijn voor het uitvoeren van economische evaluaties in de gezondheidszorg], https://www.zorginstituutnederland.nl/publicaties/publicatie/2016/02/29/richtlijn-voor-het-uitvoeren-van-economische-evaluaties-in-de-gezondheidszorg (2016).

[23] Uitdehaag B Kobelt G Berg J , et al. New insights into the burden and costs of multiple sclerosis in Europe: results for The Netherlands. Mult Scler J 2017; 23: 117–129. 10.1177/1352458517708663 28643595

[24] Sormani MP Arnold DL De Stefano N . Treatment effect on brain atrophy correlates with treatment effect on disability in multiple sclerosis. Ann Neurol 2014; 75: 43–49. 24006277 10.1002/ana.24018

[25] Dunn N Juto A Ryner M , et al. Rituximab in multiple sclerosis: frequency and clinical relevance of anti-drug antibodies. Mult Scler J 2018; 24: 1224–1233. 10.1177/1352458517720044 28762877

[26] Mao Z Álvarez-Gonzalez C De Trane S , et al. Cladribine: off-label disease modification for people with multiple sclerosis in resource-poor settings? Mult Scler J - Exp Transl Clin 2018; 4: 205521731878376. 10.1177/2055217318783767 PMC6077935 30090639

[27] Bovis F Signori A Carmisciano L , et al. Expanded disability status scale progression assessment heterogeneity in multiple sclerosis according to geographical areas. Ann Neurol 2018; 84: 621–625. 30179270 10.1002/ana.25323

[28] Kletzl H Gibiansky E Petry C , et al. N4.001 pharmacokinetics, pharmacodynamics and exposure-response analyses of ocrelizumab in patients with multiple sclerosis. Neurology 2019; 92: N4.001.

[29] Giovannoni G Popescu V Wuerfel J , et al. Smouldering multiple sclerosis: the ‘real MS’. Ther Adv Neurol Disord 2022; 15: 1–18. 10.1177/17562864211066751 PMC8793117 35096143

[30] Kappos L Wolinsky JS Giovannoni G , et al. Contribution of relapse-independent progression vs relapse-associated worsening to overall confirmed disability accumulation in typical relapsing multiple sclerosis in a pooled analysis of 2 randomized clinical trials. JAMA Neurol 2020; 77: 1132–1140. 32511687 10.1001/jamaneurol.2020.1568 PMC7281382

[31] van Kempen ZLE Toorop AA Sellebjerg F , et al. Extended dosing of monoclonal antibodies in multiple sclerosis. Mult Scler J 2022; 28: 2001–2009. 10.1177/13524585211065711 34949134

[32] Smets I Giovannoni G . Derisking CD20-therapies for long-term use. Mult Scler Relat Disord 2022; 57: 103418. 34902761 10.1016/j.msard.2021.103418

[33] Baker D Pryce G James LK , et al. The ocrelizumab phase II extension trial suggests the potential to improve the risk: benefit balance in multiple sclerosis. Mult Scler Relat Disord 2020; 44: 102279. 32645640 10.1016/j.msard.2020.102279

[34] Samjoo IA Klotz L Giovannoni G , et al. Simulated treatment comparison of efficacy outcomes for ofatumumab in ASCLEPIOS I/II versus ocrelizumab in OPERA I/II for the treatment of patients with relapsing multiple sclerosis. Mult Scler Relat Disord 2022; 66: 104031. 35841716 10.1016/j.msard.2022.104031

[35] Baharnoori M Bhan V Clift F , et al. Cost-Effectiveness analysis of ofatumumab for the treatment of relapsing-remitting multiple sclerosis in Canada. PharmacoEconomics - Open 2022; 6: 859–870. 36107307 10.1007/s41669-022-00363-1 PMC9596641

